# A neglected cervicovesical fistula diagnosed and repaired by combined hysteroscopy and laparoscopy: A case report and review of literature

**DOI:** 10.3389/fsurg.2022.986216

**Published:** 2022-11-02

**Authors:** Dongjing Sun, Wenzhi Xu, Yicheng Chen, Xueyuan Chen, Xiaona Lin

**Affiliations:** ^1^ Department of Obstetrics and Gynecology, Zhejiang University School of Medicine Sir Run Run Shaw Hospital, Hangzhou, China; ^2^ Key Laboratory of Reproductive Dysfunction Management of Zhejiang Province, Hangzhou, China; ^3^ Department of Obstetrics and Gynecology, Hangzhou Hospital of Traditional Chinese Medicine, Hangzhou, China; ^4^ Department of Urology, Zhejiang University School of Medicine Sir Run Run Shaw Hospital, Hangzhou, China; ^5^ Department of Obstetrics and Gynecology, Yiwu Shangcheng Hospital of Obstetrics & Gynecology, Yiwu, China

**Keywords:** cervicovesical fistula, hysteroscopy, intrauterine adhesions, uterus, bladder

## Abstract

**Objective:**

To analyze a case of neglected cervicovesical fistula with intrauterine adhesions caused by cesarean section.

**Methods:**

A 36-year-old female patient with a history of two previous cesarean sections complained of the absence of menstruation for the last 18 months. The diagnosis of the cervicovesical fistula was made through hysteroscopy and cystoscopy. The reconstruction of the uterus and bladder was achieved by a laparoscopic repair technique.

**Results:**

The patient resumed normal menstruation postoperatively without complaining of any complications. Uterine continuity and cavity had been restored to normal at the second look of hysteroscopy.

**Conclusions:**

Cervicovesical fistula with intrauterine adhesions is very rare in our clinical work. Hysteroscopy might play an essential role in diagnosing cervicovesical fistula and IUA. In our literature review, a surgical approach was the mainstay and definitive management of the cervicovesical fistula following a cesarean section.

## Introduction

Vesicouterine fistula is an abnormal communication between the bladder and the uterus. It represents a rare urogenital complication, accounting for approximately 1%–4% of genitourinary fistula (1). In 1908, the first case was reported by Knipe (2), and later in 1957, Youssef (3) reported on the classic symptoms of vesicouterine fistula. Risk factors include delivery in the second stage of labor, uterine rupture, placenta accrete, manual removal of the placenta, excessive intraoperative bleeding, and most history of previous cesarean section (4). Cervicovesical fistula, fistulas that form from the bladder into the cervix, is rarer still (6). We report a case of cervicovesical fistula presenting with a chief complaint of secondary amenorrhea and a review of the literature.

## Case report

A 36-year-old Chinese female patient, with a history of two previous cesarean sections, was referred to our outpatient clinic. Her chief complaint was the absence of menstruation for the last 18 months, accompanied by periodic lower abdominal distension. She said that the second cesarean section was taken emergently in a rural hospital and it was a tough procedure. She denied any allergies and had no special drug history or family history of other diseases. The physical examination at the presentation was unremarkable. A gynecological three-dimensional ultrasound examination revealed intrauterine adhesions and hydrosalpinx in the left fallopian tube.

**Figure 1:**
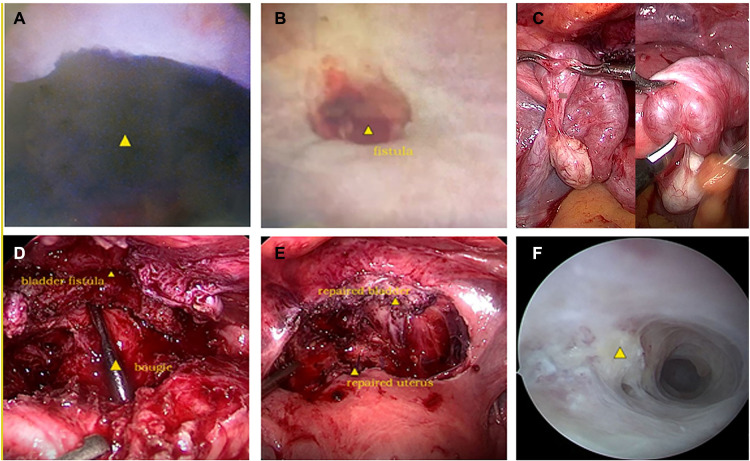
(**A**) The fistulous end of the anterior cervix in hysteroscopy; (**B**) the fistulous end of the bladder in cystoscopy; (**C**) hematosalpinx of both fallopian tubes in laparoscopy; (**D**) the identification of the fistulous end of the cervical and uterine canal by bougie, as well as bladder fistulous end in laparoscopy; (**E**) the reconstruction of the bladder and the uterus in laparoscopy; (**F**) intrauterine adhesions in hysteroscopy.

We carried out hysteroscopy examinations before the surgery. A huge defect was discovered at the 12 o'clock position in the cervical canal when the scope was moved forward (Figure 1A). Then the scope entered the bladder easily, but we could not gain access to the uterine cavity. A contracted scar filled with decayed tissue was found on the posterior bladder wall by cystoscopy (Figure 1B), measuring approximately 10 mm in diameter. The bilateral ureteral openings were normal. The lower anterior wall of the uterus was adhered to the posterior bladder wall densely by laparoscopy. Both fallopian tubes exhibited hematosalpinx (Figure 1C).

**Figure 2:**
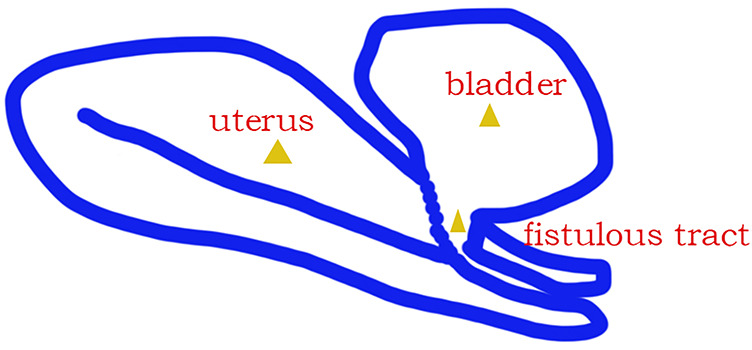
Graphic representation of the fistulous tract.

The adhesion was released carefully, and the scar was removed; finally, the fistulous tract was ended by the posterior wall of the bladder and the anterior cervix of the uterus (Figure 2) after the complete exposure of the uterus and bladder. The bladder was repaired with two layers of continuous sutures. Next, the uterus was reconstructed by two layers of interrupted sutures (Figures 1D,E). Bilateral fallopian tubal fimbriae were opened and restored. Finally, hysteroscopic adhesiolysis was conducted after the cervical defect closing. Multiple dense adhesions were observed on both sides of the uterine cavity, and the adhesions accounted for 1/3–2/3 of the area of the uterine cavity (Figure 1F). Bilateral fallopian tube openings were visible after hysteroscopic adhesiolysis.

**Figure 3:**
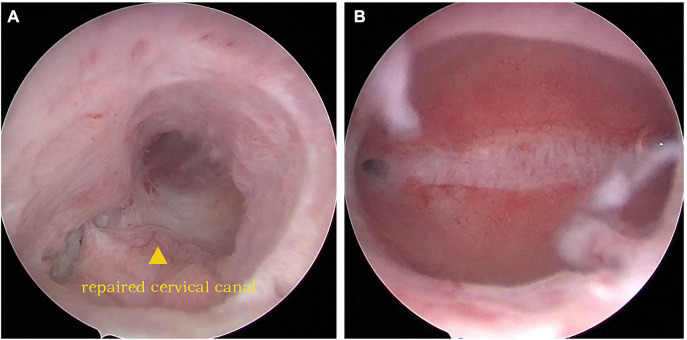
(**A**) The anastomotic stoma of the uterine isthmus and cervix; (**B**) normal uterine cavity.

No surgical complications were observed postoperatively. After the reconstructive procedure, bladder catheterization was established for one month. Furthermore, the uterine balloon was indwelled for two months. The patient could micturate normally after the catheterization was removed. Moreover, she returned to normal menstrual cycles and volume after the surgery without complaining of abnormal vaginal discharge, urinary incontinence, or menouria. The second hysteroscopy examination was carried out two months later. The uterus restored its continuity, with no new adhesion in the uterine cavity. In addition, no obstruction or adhesion was found in the cervical canal (Figure 3).

## Discussion

Cervicovesical fistula represents a rare urogenital complication. The diagnosis of cervicovesical fistula is generally based on symptoms and clinical examinations. The symptoms of cervicovesical fistula are variable. These symptoms include amenorrhea and periodic hematuria coinciding with the time of the menstrual cycle or menouria, vaginal leakage of urine, and recurrent urinary tract infections (3, 4, 5). Most cases of cervicovesical fistula result from bladder injury during lower segment cesarean section (6). The approach of sutures and suture material might also be risk factors (7). Previous cesarean section and peritoneal adhesions accounted for the main risk factors of bladder injury. Furthermore, bladder injuries may occur during different steps of the cesarean section procedure: the opening of the peritoneal cavity, the creation of the bladder flap, the hysterotomy or hysterotomy extension, and rarely the uterine suture (8). Women with a previous uterine scar are a high risk for uterine rupture during pregnancy as well (9).

For this reported case, the patient had neither the typical Youssef's syndrome nor the symptom of continuous urinary leakage. The patient chiefly complained of secondary amenorrhea after the second cesarean section. Possibly suggesting that the uterine incision was made low down in the lower uterine segment close to the cervix where the bladder could have become adherent to the scar. Due to inadequate separation of the lower uterine segment, the bladder was mistaken as the cervical side of the lower uterine segment, which was sutured with the uterine body side during that emergent procedure. The patient did not pay more attention to menstruation during lactation. With the hyperplasia of scar, the posterior uterine wall adhered to bladder resulting in outflow obstruction and amenorrhea. When the period recovered postpartum, menstrual reflux and inflammation led to atresia of the fimbria, hematosalpinx, and periodic abdominal pain.

### Diagnosis of cervicovesical fistula

Various imaging procedures have been found useful to diagnose cervicovesical fistula. Cystoscopy, cystography, hysteroscopy, and hysterosalpingography (HSG) play a crucial role. Additional imaging examinations include contrast-enhanced CT, magnetic resonance imaging (MRI), and transvaginal ultrasound (Table 1). However, the early identification of the fistula is still a challenge.

**Table 1:** Review of the literature with CERVICOVESICAL FISTULA.

Literature	Case (s)	Symptoms	Diagnostic tools	Treatments	Comments
2013 N. Rajamaheswari et al. (10)	17	Menouria or urinary incontinence or both	Intravenous urography (-); Hysterogram (-); cystogram (-); cystoscopy (+)	13 abdominal repair; 3 vaginal repair; 1 combined abdominovaginal approach	Cured
2007 J. B. Hadzi-Djokic et al. (11)	14	Vaginal urinary leakage, menouria; amenorrhea	Vaginal examination (-); cystoscopy (+); methylene blue test (+); cystography (+); hysterography (-)	5 transvesical approach; 9 transperitoneal approach with tissue-flap interposition	Cured
2018 Evangelos N et al. (14)	1	Intermittent vaginal leakage of urine	Cystoscopy (+); Vaginal ultrasound (+); contrast-enhanced CT (+)	Open repair	Cured
2017 Othman J et al. (15)	1	Hematuria	CT (+)	Robotic early repair	Cured
2021 Baker MV et al. (16)	1	Cyclical hematuria	Cystoscopy (+);	Robotic approach	Cured
2021 Achmad Kemal Harzif et al. (17)	1	Absence of menstruation cyclical haematuria,	HSG (+); cystoscopy (+); ultrasound (-)	Laparoscopic bladder fistula repair, coupled with total laparoscopic hysterectomy.	Cured
2018 Machado Junior RA et al. (18)	1	Cyclic menouria and amenorrhea	Cystoscopy (+)	The open abdominal route, coupled with hysterectomy	Cured
2020 Aly Abdel-Karim et al. (19)	14	Menouria and amenorrhea	Cystoscopy (+); retrograde cystography (+)	8 repaired by Conventional laparoscopy; 6 repaired by laparoscopic single-site surgery (LESS)	Cured

Amenorrhea is a common symptom of intrauterine adhesions, especially in severe Asherman syndrome. It was difficult to distinguish IUA from cervicovesical fistula after the second cesarean section. As a preliminary diagnosis of intrauterine adhesions, the possibility of the cervicovesical fistula was overlooked in this patient, for which the relevant imaging tests were deficient. While the application of hysteroscopy by examining the no-dilated cervical canal also played a vital role in diagnosis.

### Review of treatment for cervicovesical fistula

Treatments for cervicovesical fistula include conservative treatments and surgery. The conservative therapeutic options include bladder catheterization, hormonal therapy, and cystoscopic fulguration, all with favourable results. Nevertheless, only 5% of the cases respond to conservative therapy (10–13). Surgery is the main technique to treat cervicovesical fistula. Many different approaches have been advocated (transperitoneal, transvesical, and transvaginal), along with different surgical techniques of repair (open, conventional laparoscopy, laparoscopic single-site surgery, and robotic-assisted) (14). Most of the repairs were performed by open surgery or transvaginal access before 2015; laparoscopic repair emerged recently and soon became the major method (Table 1). Although both are considered minimally invasive, the advantage of laparoscopic surgery is a good exploration of the abdominopelvic cavity compared to the transvaginal technique, and other pelvic disorders can be treated simultaneously. In summary, hysteroscopic diagnosis and laparoscopic repair could be recommended for intricate cervicovesical fistula cases.

The surgical approach has been established as the mainstay and definitive management of the cervicovesical fistula following the cesarean section in our literature review. For this patient, the uterus had been constructed, the hematosalpinx had been cured, and the bladder had been repaired by laparoscopy. Meanwhile, intrauterine adhesions had been released by hysteroscopy. The normal shape of the uterus, uterine cavity, and bladder had been restored.

## Conclusion

Cervicovesical fistula with intrauterine adhesions is very rare in our clinical work. Therefore, it can be neglected easily. It is necessary to restore the correct anatomical structure during multiple cesarean sections, and meanwhile, early identification and diagnosis are necessary. Hysteroscopy might play an essential role in diagnosing cervicovesical fistula and IUA. Surgery is the vital approach to repairing the cervicovesical fistula. The uterus should be reconstructed as far as possible instead of hysterectomy to preserve women's reproductive function.

## Data Availability

The raw data supporting the conclusions of this article will be made available by the authors, without undue reservation.
